# Application of rapid clinical exome sequencing technology in the diagnosis of critically ill pediatric patients with suspected genetic diseases

**DOI:** 10.3389/fgene.2025.1526077

**Published:** 2025-03-10

**Authors:** Xuejun Ouyang, Dazhi Chi, Yu Zhang, Tian Yu, Qian Zhang, Lei Xu, Victor Wei Zhang, Bin Wang

**Affiliations:** ^1^ The Neonatal Intensive Care Unit, Zhujiang Hospital, Southern Medical University, Guangzhou, Guangdong, China; ^2^ Department of Emergency, Zhujiang Hospital, Southern Medical University, Guangzhou, Guangdong, China; ^3^ The Pediatric Intensive Care Unit, Hunan Provincial People’s Hospital, Changsha, Hunan, China; ^4^ Department of Genomic Medicine, AmCare Genomics Lab, Guangzhou, Guangdong, China

**Keywords:** clinical exome sequencing, mtDNA sequencing, critical illness, rapid genetic diagnosis, pediatric

## Abstract

**Purpose:**

This study evaluates the efficacy of rapid clinical exome sequencing (CES) and mitochondrial DNA (mtDNA) sequencing for diagnosing genetic disorders in critically ill pediatric patients.

**Methods:**

A multi-centre investigation was conducted, enrolling critically ill pediatric patients suspected of having genetic disorders from March 2019 to December 2020. Peripheral blood samples from patients and their parents were analyzed using CES (proband-parent) and mtDNA sequencing (proband-mother) based on Next-Generation Sequencing (NGS) technology.

**Results:**

The study included 44 pediatric patients (24 males, 20 females) with a median age of 27 days. The median turnaround time for genetic tests was 9.5 days. Genetic disorders were diagnosed in 25 patients (56.8%): 5 with chromosome microduplication/deletion syndromes (11.3%), 1 with UPD-related disease (2.3%), and 19 with monogenic diseases (43.2%). *De novo* variants were identified in nine patients (36.0%). A neonate was diagnosed with two genetic disorders due to a homozygous *SLC25A20* variant and an *MT-TL1* gene variation.

**Conclusion:**

Rapid genetic diagnosis is crucial for critically ill pediatric patients with suspected genetic disorders. CES and mtDNA sequencing offer precise and timely results, guiding treatment and reducing mortality and disability, making them suitable primary diagnostic tools.

## 1 Introduction

Congenital disabilities typically encompass congenital malformations, physiological abnormalities, and metabolic defects related to genetic, environmental, and other unidentified factors. These factors collectively constitute the primary causes of death and congenital disabilities among newborns and infants (Almli et al., 2020; World Health Organization, 2020). Various genetic diseases exist, including chromosome disorders, chromosome microduplication/deletion syndromes, single-gene genetic disorders, and mitochondrial disorders. Based on the latest statistics from the OMIM database (https://omim.org), as of 10 January 2025, the catalogue of genetic diseases exceeds 8400 entries, with new types emerging every year. Predominantly represented are single-gene genetic disorders, many of which manifest relevant clinical phenotypes early on during the neonatal and infancy stages.

Nevertheless, genetic diseases are characterized by clinical phenotypes and genetic heterogeneity. Early neonatal clinical presentations often manifest atypically, potentially overshadowed by common neonatal illnesses, or the relevant phenotypes may not have fully emerged during the neonatal or infancy phases. Certain genetic disorders advance rapidly, swiftly evolving into critical conditions. Absent timely diagnosis and targeted intervention strategies, dire consequences such as mortality and impairment may ensue. In recent years, rapid genome sequencing (rGS) based on next-generation sequencing (NGS) technology has been effectively applied to diagnose the genetic aetiology of neonatal/pediatric intensive care units (NICU/PICU) (Dimmock et al., 2020; Wu et al., 2021; Maron et al., 2023; Rodriguez et al., 2024). According to literature reports, the diagnosis reporting period of exome sequencing (ES) typically ranges from 1 to 2 weeks (Gubbels et al., 2020; Liu et al., 2021). The diagnosis reporting period of genome sequencing (GS) is usually less than 1 week, allowing prompt treatment adjustments in NICU/PICU pediatric cases (Wang et al., 2020; Sanford Kobayashi and Dimmock, 2022; Lumaka et al., 2023). This can reduce child mortality, prevent ineffective or harmful treatments, minimize unnecessary examinations, and provide prognostic information (Jezkova et al., 2022; Guner Yilmaz et al., 2024). However, the current rapid diagnosis scheme for genetic diseases generally does not include mitochondrial DNA (mtDNA) sequencing. When using ES or GS to detect mtDNA variations, the detection results of mtDNA variations may be influenced by nuclear mitochondrial DNA segments (NUMTs) in the nucleus (Xue et al., 2023). This can lead to missed diagnoses or misdiagnoses of mitochondrial diseases (Gusic and Prokisch, 2021). This study explored the applicability of clinical exome sequencing (CES) based on NGS technology and mtDNA sequencing as the first-line diagnostic technology for critically ill children with suspected genetic disorders.

## 2 Materials and methods

### 2.1 Study cohorts

The Zhujiang Hospital of Southern Medical University Ethics Committee approved this multi-centre prospective cohort study. Informed consent was obtained from the families of all children who were enrolled. From January 2020 to December 2020, this research included 44 critically ill pediatric patients with suspected genetic disorders who were admitted to Zhujiang Hospital of Southern Medical University and Hunan Provincial People’s Hospital. The inclusion criteria were: (1) Children in the NICU and PICU exhibiting one or more systemic symptoms such as unexplained dyspnea, severe uncontrollable infections, persistent jaundice, recurrent convulsions, diminished responsiveness, feeding difficulties, and abnormal muscle tone. (2) Presence of abnormal results in auxiliary examinations, such as hyperlactic acid, hyperammonemia, severe electrolyte disorder, intractable hypoglycemia, congenital heart disease, abnormal screening results of genetic metabolic diseases of blood or urine tandem mass spectrometry, and abnormal imaging and/or electrophysiology pointing to a possible genetic aetiology. (3) CES family (proband-parents) and mtDNA sequencing family (proband-mother) were selected as the first-line diagnosis methods for genetic diseases in children. Family members were informed about the testing methods, objectives, and limitations before genetic testing, and their informed consent was duly obtained. Exclusion criteria included: (1) Children who had previously undergone other genetic disease-related tests (such as karyotype analysis, chromosome microarray analysis (CMA) and ES) and whose results were positive. (2) Children with a documented family history of genetic disorders and identification of confirmed pathogenic gene variations. (3) Cases in which genetic test outcomes could not be used for familial analysis due to specific reasons. (4) Individuals with a documented history of previous trauma, exposure to drugs and toxins, or other factors that can result in organ dysfunction.

### 2.2 Clinical exome sequencing (CES)

Peripheral blood was collected from pediatric patients and their parents, anticoagulated with EDTA, and stored at 2°C–8°C. Genetic testing and family analysis were conducted using CES technology (AmCare Genomics Lab, Guangzhou, China). The genomic DNA was processed using an ultrasonic disruptor, followed by terminal repair, amplification, and purification steps to prepare the sequencing library. Specific capture probes (Roche NimbleGen, Madison, WI) were used to hybridize and enrich the DNA sequence of the target region. This region encompasses all exons of approximately 5,000 genes related to known diseases, 30bp introns upstream and downstream and known deep introns. The next-generation sequencing was performed on the MGISEQ-2000 platform of the Huada Zhizao sequencer. The MGISEQ-2000RS high-throughput rapid sequencing reagent kit was used for sequencing. The average sequencing depth of the target region reached 200×, with a coverage interval exceeding 10× accounting for 99.8% and a coverage interval surpassing 20× accounting for 99.5%. The generated sequencing reads were aligned against the human reference genome (GRCh37/hg19) using NextGENe. The analysis methods for single nucleotide variants (SNVs) and small fragment insertions and deletions (Indels) were as follows: high-frequency variations were filtered using population variation frequency databases such as dbSNP (https://www.ncbi.nlm.nih.gov/SNP/), ExAC (http://exac.broadinstitute.org), and gnomAD (http://www.gnomad-sg.org/). Pathogenic variation sites were assessed using various databases, including dbSNP, OMIM, HGMD (http://www.hgmd.org), and ClinVar (https://www.ncbi.nlm.nih.gov/clinvar/). The conservation and pathogenicity of the variations were predicted using SIFT, Polyphen2, MutationTaster, FATHMM, and other prediction software (http://159.226.67.237/sun/varcards/welcome/index). The low-quality sequencing data (with an average coverage of coding sequence less than 3×) were discarded. Copy number variations (CNVs) involving single or multiple exons were analyzed using homogenization calculation (Fromer et al., 2012). Annotations of CNVs refer to databases such as DGV (https://www.dgv/app/home), DECIPHER(https://decipher.sanger.ac.uk/), OMIM, and other databases, as well as published literature. The pathogenicity of the variation was classified according to the guidelines of the American Society of Medical Genetics (ACMG) (Richards et al., 2015; Riggs et al., 2020).

### 2.3 mtDNA sequencing

The mtDNA was sequenced using a combination of long-range PCR (LR-PCR) and NGS sequencing methods. Firstly, the full-length mitochondrial DNA (16529bp) was amplified from total genomic DNA using a one-step LR-PCR method. Subsequently, the sequencing library was prepared through a series of procedures, including ultrasonic disruption, terminal repair, amplification, and purification. The library was then sequenced on the MGISEQ-2000 platform of Huada Zhizhi Sequencer, resulting in a comprehensive coverage depth of approximately 5,000X for each coding base. Mitochondrial variations are expressed as a percentage value, which enables a quantitative assessment of changes in each base. The detection sensitivity of detecting coding region variation heterogeneity or variations load is greater than 2%. The latest Cambridge mitochondrial genome (rCRS NC_012920) was used as the reference sequence. The polymorphism of mitochondrial genome variation is classified based on data from approximately 20,000 mitochondrial genomes. Pathogenicity assessment of mitochondrial gene variations mainly refers to relevant databases, including mtDB (http://www.mtdb.igp.uu.se/), OMIM, and MitoMap (https://www.mitomap.org/MITOMAP), as well as published literature (Wong et al., 2020).

## 3 Results

### 3.1 Cohort characteristics

Forty-four critically ill children were enrolled in the study, with 39 (88.6%) inpatients in the NICU and 5 (11.4%) inpatients in the PICU. The cohort included 24 males (54.5%) and 20 females (45.5%). The median age of genetic testing was 27 days (ranging from 4 days to 13 years old), with 22 cases involving newborns (less than 28 days), 20 cases in infancy (28 days–1 year old), and 2 cases involving children (older than 1 year old).

### 3.2 Genetic disease diagnosis

CES and mtDNA sequencing were performed simultaneously, and the median turnaround time for gene detection results was 9.5 days (4∼19 days), involving Sanger sequencing verification or real-time fluorescence quantitative PCR verification (qRT-PCR). Among the 44 critically ill children suspected of having genetic disorders, 28 patients were found to have genetic variations (Table 1). Among them, 25 patients were found to have pathogenic or likely pathogenic variants, leading to a diagnosis of the corresponding genetic disorders and a positive rate of 56.8% (25/44). It is worth noting that one patient was simultaneously detected with both a variation in the *SLC25A2*0 gene (c.199–10T>G) and a mitochondrial gene variation in the *MT-TL1* (m.3243A>G). In addition, 4 cases were detected with a varitant of uncertain significance. The diagnosed genetic diseases include 1 case of chromosome microduplication syndrome (1q23.3q44 duplication, 2.3%), 4 cases of chromosome microdeletion-related disorders (9.0%), 1 case of UPD-related disorders (2.3%), and 19 cases of monogenic disorders (43.2%). *De novo* variations were identified in 9 cases (36.0%), and inherited variants were identified in 16 cases (64.0%). The 19 children were diagnosed with monogenic disorders, of which autosomal recessive (AR) cases constituted 52.6% (10 cases), followed by X-linked (XL) cases at 5.3% (1 case), autosomal dominant (AD) at 26.3% (5 cases), and a combined AR/AD instance at 15.8% (3 cases).

**TABLE 1:** Clinical phenotype and genetic variation information of twenty-eight children with detected genetic variations.

Pt	Gender	Age	Main clinical phenotype	Genes	Nucleotide/amino acid change	Zygote	Genetic origin	ACMG classification	Disease	Confirmation of genetic disorder
1	M	26 days	Seizure, coma, apnea, repeated hypoglycemia, high blood ammonia, electrolyte disorder	*SLC25A20*	c.199–10T>G	Hom	father + mother (both Het)	P	Carnitine-acylcarnitine translocase deficiency	Y
				*MT-TL1*	m.3243A>G	60.7%	mother (22.7%)	P	Mitochondrial myopathy, diabetes mellitus, deafness, etc	
2	M	8 days	Edema and shortness of breath in the whole body and limbs; Color Doppler ultrasound of urinary system: both kidneys are obviously enlarged, which is consistent with the changes of polycystic kidney	*PKD1*	c.11554delC (p.L3852Wfs*93)	Het	father (Het)	LP	Polycystic kidney disease type 1	Y
3	F	3 months	Appearance of premature infants, extremely low weight, respiratory failure, neonatal hypoproteinemia	*WAS*	c.134C>T (p.T45M)	Het	*De novo*	LP	X-linked thrombocytopenia /Wiskott-Aldrich syndrome /X-linked congenital neutropenia	Y
4	F	5 months	Respiratory distress, severe pneumonia, impaired liver function, growth retardation	*IGHMBP2*	c.1808G>A (p.R603H)	Hom	father + mother (both Het)	LP	Charcot-marie-tooth axonal 2S/distal type hereditary motor neuron disease type VI	Y
5	M	26 days	Neonatal hypoglycemia (intractable), neonatal pathological jaundice, macrosomia	*ABCC8*	c.1421A>G (p.Q474R)	Het	father (Het)	LP	Familial hyperinsulinemic hypoglycemia type 1	Y
6	F	44 days	Weak crying after birth, poor milk drinking, low muscle tone, pneumonia, binaural hearing loss, central atrial septal defect	15q11q13	>4.76 Mb Del	x1	*De novo*	P	Prader-Willi syndrome	Y
7	F	4.5 years	He began to vomit at the age of 2, and now he is admitted to hospital due to respiratory cardiac arrest and cardiopulmonary resuscitation. He has an acute onset and is in a coma, with irregular spontaneous breathing and hypothermia in his limbs.	*TANGO2*	exons 5-6 Del	x1	father (Het)+mother (x1)	LP	Recurrent metabolic encephalomyopathy crisis with rhabdomyolysis, arrhythmia and neurodegeneration	Y
					c.672C>A (p.Y224*)					
8	F	78 days	Severe pneumonia, ventricular septal defect, patent foramen ovale, slight separation of left kidney and renal pelvis, malnutrition, anemia, weight loss for more than 1 month, with cyanosis and cough.	18p11.32	>2.14 Mb Del	x1	*De novo*	LP	18p11.32 Del related disease	Y
					>52.22 Mb Dup	x3			18q12.1q23 Dup related disease	
9	M	49 days	Cardiac hypertrophy, abnormal liver function, genetic metabolism and tandem mass spectrometry showed that very long-chain acyl-CoA deaminase was deficient, and carnitine palmitoyl transferase deficiency was suspected	*SLC25A20*	c.199–10T>G	Hom	father + Mother (Het)	P	Carnitine acylcarnitine translocase deficiency	Y
10	F	49 days	Foaming at the mouth for 5 days, fever for 3 days, low birth weight, yellow skin staining, tandem mass spectrometry suggested hyperphenylalanine.	*UNC13D*	c.1055 + 1G>A	Het	Mother (Het)	P	Familial hemophagocytic lymphohistiocytosis type 3	Y
					c.2695C>T (p.R899*)	Het	father (Het)	P		
11	F	8 days	After birth, it is difficult to breathe, the right auricle is a little missing, the right eyeball is a little protruding, and the fingers cannot be straightened. After that, repeated convulsions occur, and the palm is broken, and there are many hairs on the shoulders, upper arms and thighs.	1q23.3q44	>87.01 Mb Dup	x2.4	*De novo*	LP	1q23.3q44 Dup related disease	Y
12	F	26 days	Pneumonia, hypoglycemia, hypothyroidism, hypoglycemia, epileptic dischargeetc.	*KMT2D*	c.1791_1792dupTC (p.R598Lfs*333)	Het	*De novo*	LP	Kabuki syndrome	Y
13	M	16 days	Persistent hypoglycemia after birth, feeding difficulties, neonatal infection, neonatal pneumonia, metabolic acidosis, hyperlactate, hyponatremia, hypocalcemia, jaundice, abnormal carnitine, clinically suspected hereditary metabolic disease.	*SCN1A*	c.2836C>T (p.R946C)	Het	*De novo*	LP	Generalized epilepsy with febrile seizures plus type 2 (Dravet syndrome)	Y
14	M	3 months	Postnatal hypoglycemia, low muscle tone of limbs, hyperhidrosis, irritability, shortness of breathetc.	*SLC37A4*	c.446G>A (p.G149E)	*Hem	Mother (Het)	LP	Glycogen storage disease type Ⅰb/glycogen storage disease type Ⅰc	Y
					exon4-12 Del	x1	father (x1)			
15	M	5 days	After birth, the crying is weak, cyanosis appears in the lips and extremities shortly after birth, muscle tension is low, feeding is difficult, and the auditory screen fails. EEG suggests that the brain maturity is delayed.	*CHRNB1*	c.727C>T (p.R243C)	Het	father (Het)	LP	Slow-channel congenital myasthenic syndrome type 2A/Acetylcholine receptor deficiency congenital myasthenic syndrome type 2C	Y
					c.125_128dupCGTG (p.P44Afs*29)		Mother (Het)			
16	M	24 days	Found pleural effusion for more than 17 h, diagnosed as sepsis, neonatal pneumonia, pleural effusion (bilateral), neonatal respiratory distress syndrome, hypoproteinemia and so on.	*FLT4*	c.3821A>G (p.D1274G)	Het	*De novo*	P	Hereditary lymphedema Type IA	Y
17	M	51 days	Sepsis, coagulation dysfunction, severe pneumonia, gastrointestinal bleeding, anemia, intracranial infection, patent ductus arteriosus, patent foramen ovale.	*FAH*	c.1106delG (p.G369Efs*5)	Het	father (Het)	LP	Tyrosinemia type I	Y
					c.1141A>G (p.R381G)		Mother (Het)			
18	F	18 days	Full-term cesarean section, no abnormality in prenatal examination during pregnancy, persistent hypoglycemia after birth, birth weight of 4.4kg, parents working in electroplating factory before pregnancy, and bad pregnancy history	*HNF4A*	c.323_332del10 (p.K108Sfs*15)	Het	Mother (Het)	LP	Fanconi tubular syndrome type 4/hyperinsulinemia and hypoglycemia/maturity-onset diabetes of the young	Y
19	M	3 months	Fever, refusal to milk, repeated crying, mental deterioration, past history of purulent meningitis, needle holes continued to be red and swollen after vaccination.	*TYK2*	c.209_212delGCTT (p.C70Sfs*21)	Het	father (Het)	LP	Immunodeficiency type 35	Y
					c.3239A>G (p.Y1080C)		Mother (Het)	VUS		
20	F	14 days	After birth, it was found that the blood sugar increased to 13.4 mmol/L, and the insulin level and C peptide decreased. The microarray analysis of amniotic fluid chromosome showed that the whole chromosome 6 was homozygous (AOH).	chr6	pUPD(Iso)	x2	*De novo*	P	6q24-associated transient neonatal diabetes mellitus	Y
21	M	7 days	Neonatal respiratory distress syndrome, neonatal pneumonia, premature infants, very low birth weight infants, twins.	16p13.3	>11.4 Kb Del	x1	father (x3)+mother (x1)	P	α-thalassemia	Y
22	F	17 days	Abnormal EEG, multifocal spike, convulsion, neonatal hypoxic-ischemic encephalopathy, intracranial venous sinus thrombosis, neonatal hyperbilirubinemia, septicemia	9p24.3p24.1	>8.6 Mb Del	x1	*De novo*	LP	9p24.3p24.1 Del related disease	Y
23	M	6 days	History of asphyxia rescue, weak breathing, poor response and yellow skin all over the body after birth.	*HBB*	c.-78A>G	Het	Mother (Het)	LP	β-thalassemia etc.	Y
				*G6PD*	c.1466G>T (p.R489L)	Hem	Mother (Het)	P	G6PD deficiency	
24	M	9 d	Premature infants, proteinuria 2, occult blood 2, systemic edema, low albumin, abnormal cardiac color Doppler ultrasoundetc.	*OSGEP*	c.740G>A (p.R247Q)	Hom	father + Mother (Het)	LP	Galloway-Mowat syndrome type 3	Y
25	M		Low birth weight, shortness of breath, respiratory distress, chronic bronchial dysplasia, pneumonia, anemia, patent foramen ovale, jaundice, myocardial damage, gastrointestinal bleeding, hypothyroidism, inguinal hernia.	*IFT140*	c.2495G>T (p.G832 V)	Het	Mother (Het)	VUS	Short Rib Syndrome with Polydactyly Type 9/Retinitis Pigmentosa Type 80	Y
					c.3873 + 1G>A		father (Het)	LP		
26	F	4 years	Amniotic fluid reduction detected at 7 months of pregnancy, infantile spasms diagnosed at 3 months old, nephrotic syndrome confirmed around 2 years old, with recent increase in seizure activity followed by fever and oliguria.	*NUP107*	c.460A>G (p.M154 V)	Het	father + Mother (Het)	VUS	Nephrotic Syndrome Type 11/? Ovarian Dysgenesis Type 6/Galloway-Mowat Syndrome Type 7	N
					c.1085C>T (p.A362V)					
27	M	13 years	Recurrent rashes for 12 years, with hypokalemia, elevated lactate, mild to moderate anemia, negative for lupus erythematosus, and liver function showing decreased albumin.	*HLCS*	c.1544G>A (p.S515N)	Hom	father + Mother (Het)	VUS	Deficiency of pyruvate carboxylase	N
28	M	37 days	Preterm birth, low tyrosine in dried blood spot, suspected metabolic disorder, TSH >100, T3 0.97 nmol/L, T4 51.5 nmol/L, vomiting after breakfast, currently on infant formula.	22q11.21	>1.35 Mb Dup	X4	father (X3)	VUS	22q11.2 Dup related disease	N

Patient, P; M, male; Female, F; y, years old; m, months; d, days; Hom, homozygote; Het, heterozygote; Hem, hemizygote; Dup, duplication; Del, deletion; P, pathogenic; LP, likely pathogenic; VUS, variant of uncertain significance; Yes, Y; No, N.

### 3.3 Cases of special genetic diseases

Case 1, a 28-day-old infant, presented with seizures, coma, respiratory arrest, recurrent hypoglycemia, hyperammonemia, electrolyte imbalances, and neutropenia. Genetic analysis revealed a homozygous variation in the *SLC25A20* gene (c.199–10T>G), leading to carnitine-acyl carnitine transferase deficiency. Additionally, a mitochondrial gene variation in *MT-TL1* (m.3243A>G) was identified, with a mutation load of 60.7% and a maternal contribution of 22.7%. These variations were classified as pathogenic, resulting in diagnoses of both carnitine-acyl carnitine transferase deficiency and mitochondrial disease.

An infant female (case 20), 14 days old, presented with postnatal hyperglycemia reaching up to 13.4 mmol/L, diminished insulin and C-peptide levels. SNP analysis found that the proband has paternal isodisomy of the entire chromosome 6 (pUPD, Isodisomy). Additionally, the analysis of polymorphic sites in the relevant exonic regions confirmed the paternity within the family. This genetic condition is notably linked to 6q24-related transient neonatal diabetes mellitus (TNDM), which presents with a distinct absence of ketosis despite elevated blood sugar levels and may be accompanied by a range of developmental issues. Ultimately, the infant was diagnosed with 6q24-associated TNDM.

**FIGURE 1:**
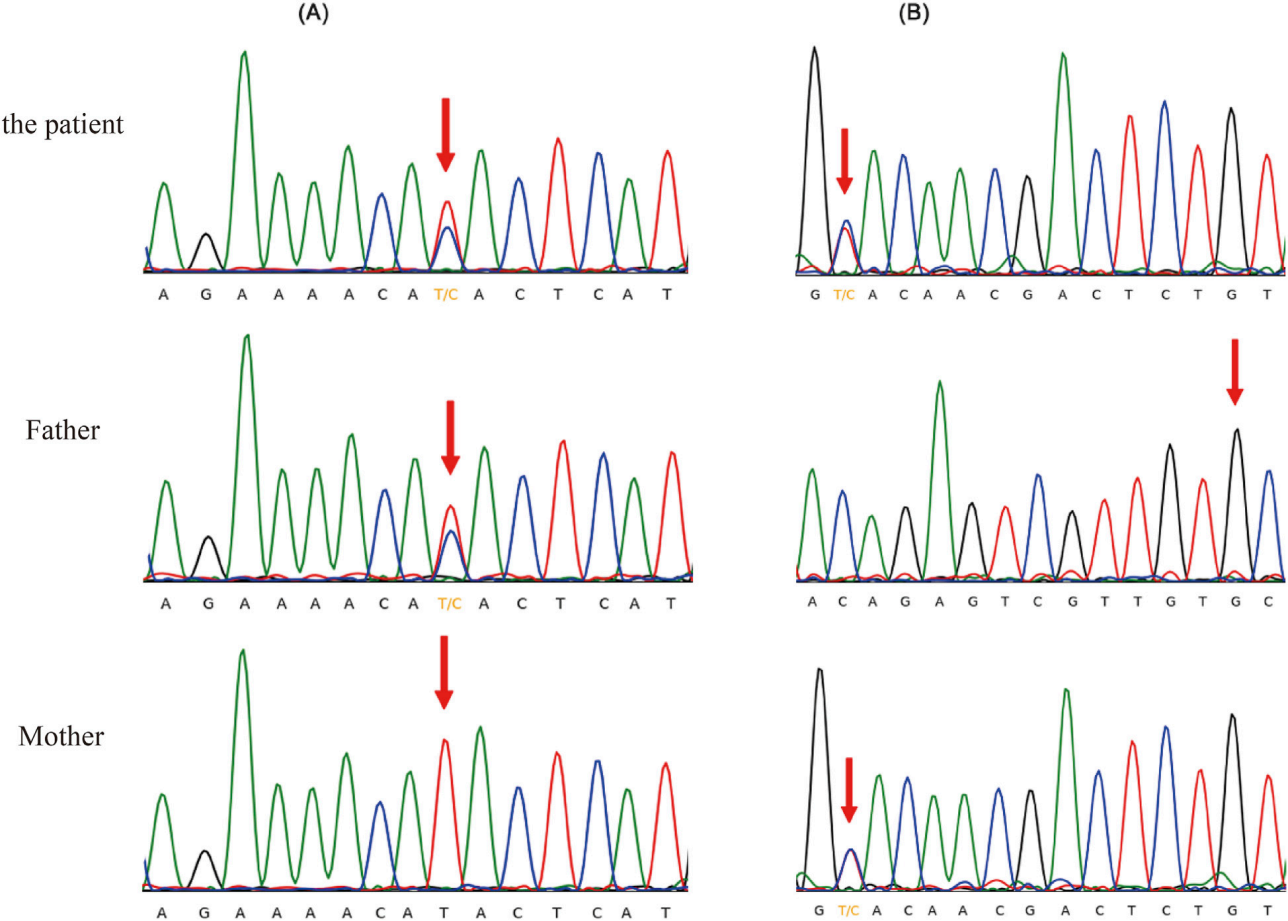
Sanger sequencing validation of the heterozygous variants in the *NUP107* gene. **(A)** c.460A>G (p.M154V), **(B)** c.1085C>T (p.A362V).

Moreover, a 4-year-old female child (case 26) presents with unexplained massive proteinuria, hypoproteinemia and oedema. When the mother was 7 months pregnant, the ultrasound imaging indicated decreased levels of amniotic fluid. Subsequent CES identified complex heterozygous variants c.460A>G (p.M154 V) and c.1085C>T (p.A362 V) of the *NUP107* gene in the children. These variants were inherited from the child’s father and mother (both heterozygous) and were subsequently validated through Sanger sequencing (Figure 1). Notably, these two *NUP107* gene variants are novel, as they have not been previously reported in related clinical cases. Their occurrence is infrequent within the gene database of the reference population. The computer-aided analysis predicts that these two variants may affect the structure or function of the protein. According to the American ACMG variation classification guide, these two variations are categorized as “of uncertain significance.”

## 4 Discussion

Hereditary disorders are the primary cause of mortality in children admitted to NICU/PICU and significantly contribute to infant mortality (Cunniff et al., 1995; Kingsmore et al., 2024). It is well-recognized that most genetic disorders exhibit clinical phenotypes during the neonatal period or early infancy, including conditions associated with variations in mitochondrial DNA (Ebihara et al., 2022; Zeviani and Viscomi, 2022). However, diagnosing the condition can be challenging due to atypical or early-stage clinical manifestations during the neonatal period. Furthermore, some severe disorders manifest rapidly, leading to potentially life-threatening situations. Thus, it is imperative to have an expedient and accurate clinical diagnosis in order to guide treatment decisions and prognostic assessments, thereby reducing disability and mortality rates.

In this prospective multi-centre study, we examined 44 critically ill children from NICU/PICU suspected of having genetic disorders using rapid CES, mtDNA sequencing, and family analysis to elucidate potential genetic etiologies. Among them, 25 children received diagnoses of genetic disorders, resulting in a positivity rate of 56.8%. This group included 5 cases of CNV microdeletion or microduplication-related disorders, 1 case of UPD-related disorder, and 19 cases of monogenic genetic disorders. Notably, one newborn was diagnosed with both a monogenic genetic disorder (*SLC25A20* gene variation) and a mitochondrial disorder (m.3243A>G). Among the children diagnosed with hereditary disorders, *de novo* variations were identified in 36.0% (9/25) of the cases. In addition to identifying pathogenic variants directly linked to the patients’ clinical conditions, genetic testing also has the potential to reveal secondary and incidental findings (SFs and IFs). SFs were defined as clinically significant variants that are actively searched for during sequencing, independent of the patient’s primary indication for testing, while IFs referred to variants that are unintentionally discovered and unrelated to the clinical presentation (Saelaert et al., 2018; Miller et al., 2023). In this study, no SFs or IFs were detected. The identification and management of SFs and IFs remain critical aspects of clinical genomics, particularly as WGS and expanded exome sequencing strategies become more prevalent. Current guidelines emphasized that patients and their families should have the opportunity to opt in or opt out of SF disclosure before testing (Miller et al., 2023). If broader genomic testing approaches, such as WGS, are incorporated into future studies, establishing a structured framework for handling SFs and IFs will be essential. This includes implementing comprehensive informed consent processes, developing standardized disclosure protocols, and ensuring access to genetic counseling to help patients and their families navigate the potential implications of unexpected findings. The ethical, psychological, and clinical considerations associated with SFs and IFs must be carefully balanced to maximize the clinical utility of genomic data while respecting patient autonomy. Future research should further explore best practices for managing these findings in critically ill pediatric patients, integrating advancements in variant interpretation and ethical guidelines to optimize patient care (Saelaert et al., 2018).

NGS technology has become an essential tool for clinicians to diagnose genetic disorders. These methods can be divided into WGS, ES, and targeted sequencing (like CES). The human genome comprises the nuclear genome, which consists of approximately 3 billion base pairs, and the mitochondrial genome, which comprises 16,569 base pairs. Within this genome, all exons, which are protein-coding regions, constitute merely 1%–2% of the total, yet they encompass around 85% of disease-related variations. WGS sequences the whole genome’s bases, including nuclear and mitochondrial genomes. On the other hand, WES is used to sequence the exon regions of over 20,000 known genes. Targeted sequencing is primarily used to sequence specific genes, typically known as pathogenic genes or genes of interest. For instance, CES encompasses all disease-related genes known in the OMIM database. Because the reporting period of conventional NGS-based genetic testing spans 4–6 weeks, it is unsuitable for diagnosing genetic disorders in critically ill or progressively deteriorating children. In recent years, rGS based on NGS technology has been effectively applied to diagnose suspected genetic disorders among children in NICU/PICU settings. Rapid exon sequencing, such as ES, offers diagnosis reports within 1–2 weeks, while the diagnostic reporting time for fast GS is usually under 1 week. In some cases, the preliminary test results can be available within 24 h (Clark et al., 2019), thereby promptly influencing children’s treatment and management in the NICU/PICU. Compared to ES, GS eliminates the need for probe capture in targeted regions, resulting in a shorter reporting period. Consequently, it is the most widely used method for rapidly diagnosing genetic disorders. In this study, the median time to obtain gene detection results through rapid CES and mtDNA sequencing was 9.5 days, ranging from 4∼19 days, including the time required for Sanger sequencing verification or qRT-PCR validation.

The results of several cohort studies on rapid diagnosis of genetic causes indicate that the positive rate of rGS in critically ill children is 30%∼69.7% (van Diemen et al., 2017; Farnaes et al., 2018; Sanford et al., 2019). Similarly, the positive yield of rapid ES is 45%∼72.2% (Bourchany et al., 2017; Lunke et al., 2020; Śmigiel et al., 2020; Wang et al., 2020), encompassing both individual proband and family-based examinations and analyses. In a cohort study, rapid CES, encompassing 4,503 known disease-associated genes, was applied to analyze a group of 20 children from the NICU/PICU/CICU, yielding a positive diagnostic rate of 50% (Brunelli et al., 2019). This study used rapid CES and mtDNA sequencing as the first-line diagnostic methods for family analysis. Custom capture probes were designed to target all exons, 30-base pair introns, and well-recognized deep introns within approximately 5,000 disease-associated genes catalogued in the OMIM database. The positive diagnostic rate for gene-related disorders was 56.8% (25/44). Strict inclusion criteria are crucial for improving the diagnostic yield of genetic testing, as narrowing the criteria to individuals with clearly defined phenotypes significantly results in more consistent estimates (Dellava et al., 2011). Studies have demonstrated that applying well-defined criteria, such as family history, clinical symptoms, or age, improves variation detection rates and diagnostic yields across various genetic disorders, including monogenic kidney diseases (Vaisitti et al., 2021), cerebral cavernous malformations (Spiegler et al., 2014), and neurofibromatosis type 1 (Castellanos et al., 2020; Kehrer-Sawatzki and Cooper, 2022). Especially, vaisitti et al. demonstrated that the implementation of strict inclusion criteria in CES greatly improved diagnostic outcomes in monogenic kidney diseases (Vaisitti et al., 2021).

WGS is the sequencing of all bases in the whole genome, which can detect SNV, Indels, and CNVs in all exon regions and include the variation of intron region and non-coding region, genome structure variation (SV), and partial mtDNA variations (Willig et al., 2015). Consequently, WGS may have more advantages in detecting and analyzing different variations. However, the cost of WGS is substantial, and numerous variations are detected, including variations in non-coding regions and deep introns, as well as a significant number of small fragment deletions/repetitions. Because it is difficult to judge pathogenicity without the support of a database, the available information is limited, which hinders its usefulness in clinical disease elucidation. Therefore, the diagnostic positive yield of WGS does not significantly surpass that of ES. Concerning genetic disease diagnosis, ES primarily detects and analyzes the SNV and Indels variations in the exon region of nuclear genes. This technique is utilized to diagnose monogenic diseases. Combined with complementary methodologies for CNV detection, such as CMA, it generally meets most clinical diagnostic requirements. Notably, the average sequencing depth of ES typically reaches around 100×. Previous studies have demonstrated that read-depth analysis can detect copy number variations involving more than three exonic fragments (Hehir-Kwa et al., 2015; Retterer et al., 2015). In this study, the average sequencing depth of CES is approximately 200×. The homogeneous algorithm can effectively analyze CNVs and detect single exon deletions (case 13, case 20). Moreover, it simultaneously analyzes diverse variation types variations, including SNVs, Indels, and CNVs. This approach’s primary focus is the evaluation of approximately 5,000 genes associated with known disorders. The interpretation of variation pathogenicity, accuracy, periodicity, and cost aligns well with the requirements of rapid diagnosis for clinical genetic disorders.

The human mitochondrial genome is a circular, double-stranded DNA molecule comprising 16,569 base pairs and 37 genes. These genes include two rRNA (12S, 16S), 22 tRNA, and 13 polypeptides related to oxidative phosphorylation. The main types of mtDNA variations are point variations and recombination variations. Due to factors such as variation load, tissue distribution, organ-specific reliance on the respiratory chain, and environmental influences, the clinical manifestations of mitochondrial diseases are complex and diverse. These manifestations range from dysfunction in a single organ to multi-system, making it often challenging to differentiate from other diseases (Orsucci et al., 2021). Molecular genetic testing is crucial for neonates suspected of mitochondrial diseases (Mady et al., 2024), as a clear diagnosis can provide targeted treatment for some patients, alleviating their condition. Common genetic testing methods include Sanger sequencing and NGS, which encompasses targeted panel testing, WES, and WGS. According to the Expert Consensus on the Diagnosis and Treatment of Neonatal Mitochondrial Diseases (2024) (Jun et al., 2024), when a neonatal mitochondrial disease is highly suspected based on clinical phenotype, targeted testing for common mtDNA and/or nDNA mutations can be performed. For critically ill neonates who require comprehensive testing for mitochondrial-related diseases, and due to the atypical clinical phenotype and the fact that approximately 80% of pediatric mitochondrial diseases are caused by nDNA mutations (Gusic and Prokisch, 2021), the first choice is rapid WES combined with mtDNA testing, with WGS performed when necessary. In this study, the sequencing of mtDNA based on LR-PCR and NGS can effectively address the interference issues of SNVs and CNVs arising from NUMTs. This approach ensures accurate identification of mtDNA variations and their corresponding variation load. In a newborn, a variation load of 60.7% was observed in the *MT-TL1* gene, with 22.7% being inherited maternally. This clear genetic basis facilitates the diagnosis of mitochondrial disease. Additionally, the concurrent deficiency of carnitine-acyl carnitine translocation deficiency in the child somewhat obscures the clinical phenotype of mitochondrial disease. Consequently, continuous monitoring and appropriate interventions are imperative to improve prognosis.

However, in our study, we did not systematically track the follow-up of cases without a genetic diagnosis. Future research would collect more comprehensive follow-up data, including the use of WGS or other diagnostic methods, to assess how these approaches may improve diagnostic yields when CES and mtDNA sequencing fail to provide conclusive results.

## 5 Conclusion

In summary, rapid genetic diagnosis is highly valuable for critically ill pediatric patients suspected of having genetic diseases. It can guide clinical treatment strategies and prognostic assessments in confirmed cases, effectively reducing mortality and disability rates. Furthermore, it establishes a foundation for future genetic counselling regarding fertility-related considerations. For cases lacking definitive diagnoses, the outcomes of genetic testing remain significant for differential diagnosis and guiding therapy. Implementing rGS in clinical practice must prioritize clinical diagnosis and provide practical solutions to patient dilemmas. The precision, regularity, and comprehensibility of results obtained from rapid CES and mtDNA sequencing in this study align with the requirements of clinical diagnosis. Thus, they can be adopted as the primary detection technique for critically ill children who are suspected of having genetic diseases.

## Data Availability

The datasets presented in this study can be found in online repositories. The names of the repository/repositories and accession number(s) can be found in the article/Supplementary Material.
