# Corrigendum: Application of rapid clinical exome sequencing technology in the diagnosis of critically ill pediatric patients with suspected genetic diseases

**DOI:** 10.3389/fgene.2025.1592212

**Published:** 2025-04-11

**Authors:** Xuejun Ouyang, Dazhi Chi, Yu Zhang, Tian Yu, Qian Zhang, Lei Xu, Victor Wei Zhang, Bin Wang

**Affiliations:** ^1^ The Neonatal Intensive Care Unit, Zhujiang Hospital, Southern Medical University, Guangzhou, Guangdong, China; ^2^ Department of Emergency, Zhujiang Hospital, Southern Medical University, Guangzhou, Guangdong, China; ^3^ The Pediatric Intensive Care Unit, Hunan Provincial People’s Hospital, Changsha, Hunan, China; ^4^ Department of Genomic Medicine, AmCare Genomics Lab, Guangzhou, Guangdong, China

**Keywords:** clinical exome sequencing, mtDNA sequencing, critical illness, rapid genetic diagnosis, pediatric

In the published article, there was an error in the **Author List**, and author Dazhi Chi was erroneously [excluded]. The corrected author list appears above.

In the published article, there was an error in the **Author contributions** statement. Author Dazhi Chi contributions were erroneously excluded. The corrected statement appears below.

“XO: Data curation, Writing – original draft. DC: Conceptualization, Investigation, Methodology, Writing – review and editing. YZ: Formal Analysis, Writing – review and editing. TY: Data curation, Writing –review and editing. QZ: Methodology, Writing – review and editing. LX: Methodology, Writing – review and editing. VZ: Methodology, Writing – review and editing. BW: Funding acquisition, Supervision, Writing – review and editing.”

The authors apologize for this error and state that this does not change the scientific conclusions of the article in any way. The original article has been updated.

