# Corrigendum: Corrigendum: Application of rapid clinical exome sequencing technology in the diagnosis of critically ill pediatric patients with suspected genetic diseases

**DOI:** 10.3389/fgene.2025.1620297

**Published:** 2025-05-12

**Authors:** Xuejun Ouyang, Dazhi Chi, Yu Zhang, Tian Yu, Qian Zhang, Lei Xu, Victor Wei Zhang, Bin Wang

**Affiliations:** ^1^ The Neonatal Intensive Care Unit, Zhujiang Hospital, Southern Medical University, Guangzhou, Guangdong, China; ^2^ Department of Emergency, Zhujiang Hospital, Southern Medical University, Guangzhou, Guangdong, China; ^3^ The Pediatric Intensive Care Unit, Hunan Provincial People’s Hospital, Changsha, Hunan, China; ^4^ Department of Genomic Medicine, AmCare Genomics Lab, Guangzhou, Guangdong, China

**Keywords:** clinical exome sequencing, mtDNA sequencing, critical illness, rapid genetic diagnosis, pediatric

In the published article, it was erroneously excluded that Authors Xuejun Ouyang and Dazhi Chi contributed equally to the work and share first authorship.

The authors apologize for this error and state that this does not change the scientific conclusions of the article in any way. The original article has been updated.

